# How Reproductive Ecology Contributes to the Spread of a Globally Invasive Fish

**DOI:** 10.1371/journal.pone.0024416

**Published:** 2011-09-19

**Authors:** Amy E. Deacon, Indar W. Ramnarine, Anne E. Magurran

**Affiliations:** 1 Scottish Oceans Institute, School of Biology, University of St Andrews, Fife, Scotland, United Kingdom; 2 Department of Life Sciences, University of the West Indies, St Augustine, Trinidad and Tobago; Institute of Marine Research, Norway

## Abstract

Invasive freshwater fish represent a major threat to biodiversity. Here, we first demonstrate the dramatic, human-mediated range expansion of the Trinidadian guppy (*Poecilia reticulata*), an invasive fish with a reputation for negatively impacting native freshwater communities. Next, we explore possible mechanisms that might explain successful global establishment of this species. Guppies, along with some other notable invasive fish species such as mosquitofish (*Gambusia* spp.), have reproductive adaptations to ephemeral habitats that may enable introductions of very small numbers of founders to succeed. The remarkable ability of single pregnant guppies to routinely establish viable populations is demonstrated using a replicated mesocosm set up. In 86% of cases, these populations persisted for two years (the duration of the experiment). Establishment success was independent of founder origin (high and low predation habitats), and there was no loss of behavioural performance amongst mesocosm juveniles. Behavioural “signatures” of the founding locality were, however, evident in mesocosm fish. Our results demonstrate that introductions consisting of a single individual can lead to thriving populations of this invasive fish and suggest that particular caution should be exercised when introducing this species, or other livebearers, to natural water bodies.

## Introduction

It is widely recognised that invasive species represent a major threat to biological diversity [1], [2], [3]. Although impacts have been documented across terrestrial and marine systems, freshwater fish assemblages appear particularly vulnerable to the presence of exotics. Indeed, sixty-eight percent of 20^th^ century fish extinctions in North America are associated with introduced species [4] and model predictions suggest that introductions of exotic species will continue to pose a serious threat to natural communities [5].

Invasive species impact biological diversity in two ways; that is through reductions in the variety and abundance of species at a defined locality and also through reductions in the distinctiveness of assemblages at different localities. The former occurs when invasive species increase extinction rates amongst native species or lead to reductions in the size of local populations. A classic example is that of the Nile perch invasion in Lake Victoria. While the pre-invasion ecosystem supported over 400 fish species, by the end of the 20^th^ century the lake was dominated by just three – only one of which was indigenous [6]. However the distinctiveness of assemblages at different localities is also diminished by invasive species. Fish faunas become homogenized when the same species invade multiple assemblages [7]. In the United States, a pairwise comparison revealed a considerable increase in fish fauna similarity between states since European settlement – a mean of 15 more species in common per pair of states [8]. Over evolutionary time the heterogeneity and isolation of freshwater habitats has contributed to the diversity of freshwater fish [9], which make up around 43% of the estimated 32,500 species of fish on Earth [10], even though freshwater accounts for <0.01% of water on the planet [11]. From a global perspective, therefore, increased homogeneity is associated with marked transformations of freshwater communities. It means that the same subsets of invasive species will increasingly be found in geographically separated freshwater systems that historically supported distinct communities of fish.

The erosion of biological diversity poses significant challenges for scientists and managers. It is essential on one hand to document range expansion in the species that are most implicated in biotic homogenization and on the other to understand the mechanisms that enable these taxa to establish viable populations following accidental or deliberate introduction. The Allee effect means that colonizing populations below a minimum number of founders are less likely to become established [12]. Tobin *et al.* [13] found that the invasion speed of the gypsy moth in North America tended to be slower in regions where more founders were needed to establish a population, suggesting that mechanisms that enable species to establish at small propagule size may play a key role in successful invasions. The minimum propagule size is a single individual. In sexual species this means a single pregnant female.

The Trinidadian guppy, *Poecilia reticulata*, is now recognised as an invasive that negatively impacts native fish assemblages [14]; it is also a species with the potential to establish at small propagule size. The guppy's native range is Trinidad and Tobago, and the coastal zone of NE South America [15] but this has been vastly extended as a result of human intervention and the species is now widely distributed in tropical freshwaters [16]. Here we integrate information from a new survey of fish biologists worldwide, with existing reports on guppy distribution, to produce the most complete picture of the current distribution of this invasive species to date, and show that it is contributing to the homogenisation of fish communities on a global scale.

We then examine the capacity of the species to form viable populations in novel environments. Trinidadian guppies belong to a group of fish characterised by ovoviviparity and sperm storage [17] and single females can give birth to multiple broods of live offspring [18]. Sperm are stored for up to six months, and broods may be fathered by several males [19], [20]. Guppies naturally occur in ephemeral or isolated habitats where females may have limited opportunities of encountering a mating partner [15]. Sperm storage, combined with live birth, is advantageous in these circumstances but may also pre-adapt these fish for invasive success. To date there is one documented case of a single guppy successfully founding a population [21]. Thus, while single females clearly have the potential to establish viable populations it is not known whether this is a routine event.

There are two ways in which humans can introduce guppies – either accidentally or deliberately – into new environments, and in both cases these are likely to involve very few or even single individuals. The first route is the now well-established practise of placing guppies in water tanks and other small bodies of water as a means of controlling mosquitoes. This method was favoured by the British Colonial Administration in the early part of the 20^th^ century, and resulted in the spread of guppies across the British Empire [22], [23]. The same approach to mosquito control continues to be championed today. For example, in the state of Karnataka, India, guppies introduced to village wells and troughs appear to be effective at eradicating malaria [24]. Crucially, a single fish is sufficient for effective mosquito control in these small containers (IWR pers. obs.). Moreover, water containers are prone to flooding during the rainy season with the result that the fish they house can be released into natural drainage systems. Thus, if the single females employed in mosquito control are consistently able to found viable populations of guppies in these sorts of small, and otherwise fish free environments, natural communities will be vulnerable to repeated invasions of exotics.

The second route through which guppies are introduced is by fish hobbyists who either accidentally or deliberately release ornamental fish [16], [25], [26]. Here again any releases are likely to consist of very small numbers of individuals.

Trinidadian guppies have the status of a model species in evolutionary ecology and provide text book examples of evolution in action [15]. Natural populations of the species in Trinidad exhibit considerable geographical variation in behavioural and life history characteristics, primarily linked to variation in predation regime [27]. Fish that coexist with predators have more pronounced antipredator behaviours [28]. Life history strategies also vary. Guppies that occur in localities where there are high levels of predation tend to mature faster and invest in more, smaller offspring than those that have evolved under ‘low predation’ regimes [29]. This contrast in reproductive potential means that the likelihood of a single pregnant female establishing a viable population may depend on her origin. Specifically, the invasiveness of females derived from localities where there are many predators may be greater than those originating from low risk sites.

This study has twin aims. The first is to document the current global distribution of the guppy, collating information on the extent of its range, the primary routes of introduction and reported impacts. By doing this we show the extent to which this species is contributing to the homogenization of fish faunas at a global level. The second aim is to test the prediction that single pregnant female guppies routinely establish viable populations – that is whether the accidental or deliberate release of a single individual female is likely to result in a successful founder event. Our primary measure of viability is a self-sustaining population that persists for at least a year. We also compare the performance of newborn fish in the newly-founded populations with those from wild caught controls as an additional measure of viability. The focus on newborn fish is important as poeciliids can be highly cannibalistic, and populations will not establish if juveniles are unable to escape predatory attacks from older conspecifics [30]. Performance is a composite measure based on schooling behaviour, evasion ability, time spent in cover, activity and reaction distance. Given the natural variation in life history traits, we further ask whether fish origin affects invasion potential. Here we test the prediction that successful populations establish at a reduced rate when females originate from localities where there is a low natural risk of predation. Our experiment compares fish from two Trinidadian localities, the Upper Tunapuna and Lower Tacarigua Rivers, which are well-documented examples of low-predation and high-predation localities respectively. We examine these questions using a replicated mesocosm setup at the University of the West Indies (UWI), Trinidad & Tobago.

## Results

### I. Worldwide survey

#### Distribution and origins

**Figure 1:**
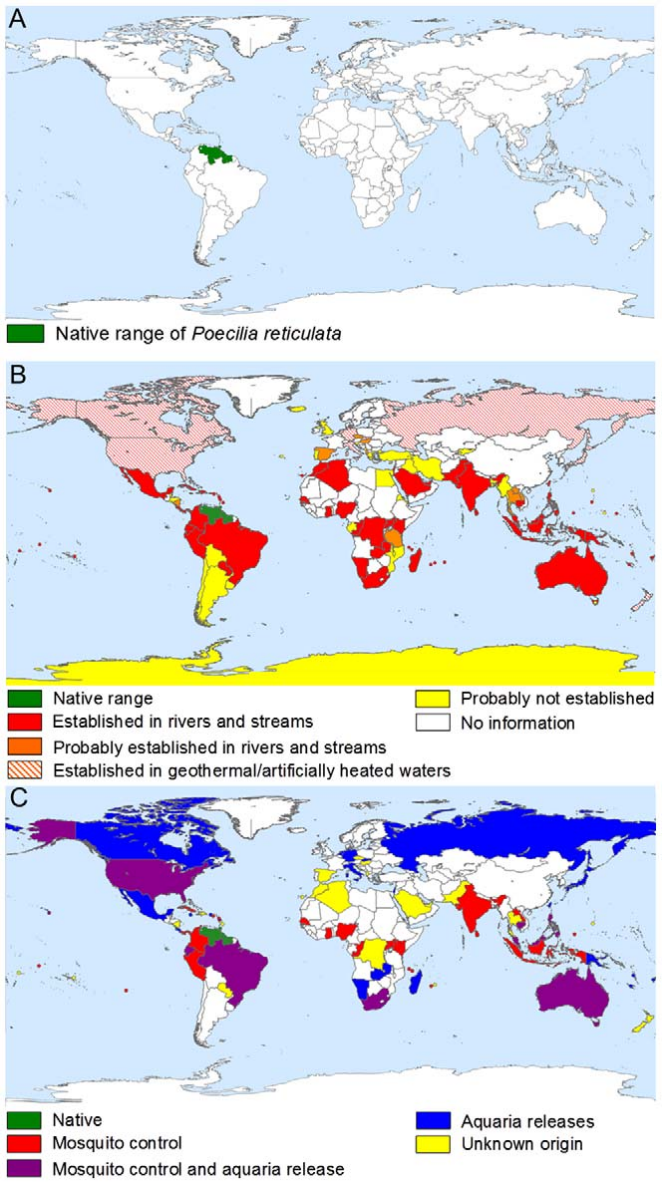
Global distribution and origins of *Poecilia reticulata* including (A) their native distribution compared with (B) their distribution as a result of introductions and (C) reported reasons behind introductions. Includes records from online databases and published literature, in combination with questionnaire responses. Countries are filled on the basis of reports from at least one location within the country and it should not be inferred that guppies are necessarily present or absent throughout. Coloured dots have been used where necessary to represent data for small islands. Maps were constructed using Manifold (v.8).

The distribution of the guppy has expanded dramatically (Figure 1 A, B). It is now established in at least 69 countries outside of its native range (see Table S1 for list of countries).

Questionnaire responses suggested that in approximately 41.5% of cases where information on origin is available, introduction can be attributed to mosquito control alone. A further 41.5% can be attributed to accidental release of aquaria fish and in around 17% of cases, a combination of both mosquito control and aquaria releases appear to be responsible for the presence of guppies (Figure 1 C).

**Figure 2:**
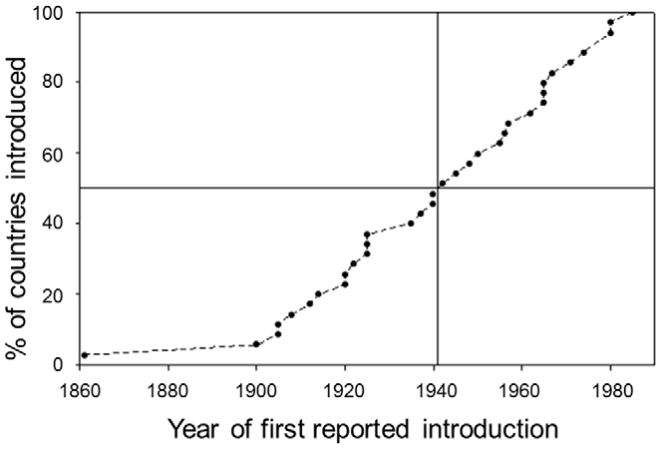
Cumulative percentage of countries subject to guppy introductions over time. Only those countries for which the date of first introduction is known are included. Gridlines indicate date by which 50% of these countries had been subject to their first guppy introduction.

Information on the date of first introduction was available for a total of 36 out of the 72 countries where guppies have been reported as definitely or probably established (see Table S1). Of the countries for which a date of introduction is available, 50% had an introduction of guppies before 1941. Between 1900 and 1985 the rate of introductions appears to have been reasonably constant (Figure 2).

#### Impacts

The reports of negative impacts of guppies include their implication in the decline of certain native species, both through the spread of disease and competition for resources; they are also associated with more general ecosystem level effects and in a few cases are reported to interfere with aquaculture processes (Table S2).

### II. Mechanisms of establishment

#### Establishment success

Two fish from the initial thirty tanks died within the first week of the experiment, were recorded as ‘extinctions’ and promptly replaced. 91% of mesocosm populations persisted at the end of year one; 86% at the end of year two.

**Figure 3:**
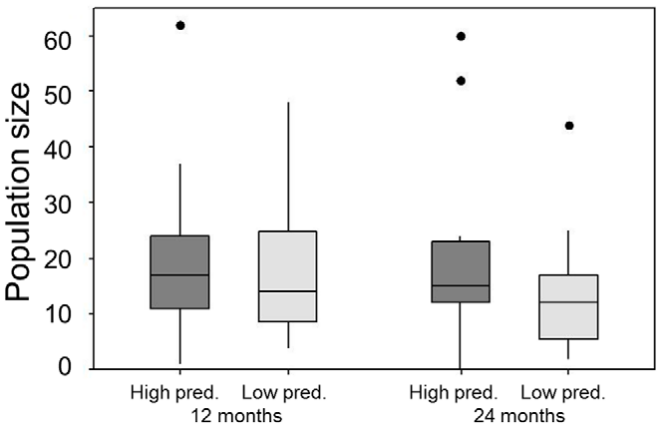
Population size of mesocosms. Numbers of individuals in populations founded by females originally from high predation and low predation localities after both 12 and 24 months. Medians, interquartile ranges and outliers (dots) are shown. N = 16 except ‘low predation, 24 months’, where N = 13.

There was no significant difference in population size between those founded by females from low or high predation populations (F_1,51_ = 0.667; p = 0.418) (Figure 3).

Newborns were recorded in all tanks by eight weeks post-introduction. There was a significant difference in mean size of first brood between the two conditions, which was larger for females originating from high predation populations (high predation origin: 13±1.66SE; low predation origin: 8.6±0.99SE; t = 2.27; df = 28; p = 0.031).

#### Newborn performance

No improvement in experimenter catching ability was apparent when escape times were plotted against testing order (Pearson's correlation = 0.056; p = 0.449) (see Figure S1).

There was no significant difference between the behavioural performance of offspring born to mesocosm or wild fish. There was a significant effect of evolutionary history, with offspring born to descendants of low predation fish displaying greater evasion ability and reaction distance (Table 1).

**Table 1:** MANOVA analysis of behavioural performance.

	Wilk's λ	df	F	P
*Origin of mother (wild or mesocosm)*	0.851	5,47	1.642	0.168
schooling		1,51	3.221	0.079
evasion		1,51	1.247	0.269
time in cover		1,51	2.209	0.143
activity		1,51	0.423	0.518
reaction		1,51	0.556	0.459
*Evolutionary history (high predation or low predation)*	0.700	5,47	4.020	0.004^b^
Schooling		1,51	0.305	0.583
evasion		1,51	6.319	0.015^a^
time in cover		1,51	0.036	0.849
activity		1,51	2.304	0.135
reaction		1,51	7.134	0.010^b^

Origin of mother and evolutionary history are included as fixed factors.

a significant at the 5% level;

b significant at the 1% level.

**Figure 4:**
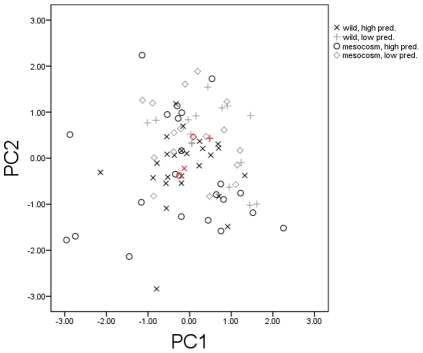
Scores generated by the behavioural performance PCA. Plotted according to the first two principal components. Red symbols represent group means.

The first principal component (PC1) explained 34% of the variation, and PC2 explained a further 26%. Higher values of PC1 were positively associated with activity and reaction distance, whilst higher values of PC2 were positively associated with time in cover and evasion ability (Figure 4).

There was no significant difference in PC1 scores between mesocosm and wild populations (F_1,75_ = 1.125; p = 0.292) or between high and low predation origins (F_1,75_ = 3.187; p = 0.078). PC2 scores also did not differ significantly between mesocosm and wild populations (F_1,75_ = 0.025; p = 0.876) but, like the MANOVA, indicated a significant effect of original locality (F_1,75_ = 11.904; p = 0.001).

## Discussion

Our results demonstrate the invasiveness of the guppy, and reveal a mechanism that has aided its dramatic range expansion.

### I. Worldwide survey

Despite the native range of this species being confined to an approximately 10° latitudinal range just north of the equator in Trinidad and Tobago and the north-eastern coastal margins of Venezuela, Guyana and Surinam, the guppy's introduced range spans every continent with the exception of Antarctica, as well as numerous oceanic islands. These new data show that the worldwide distribution of the guppy is considerably more extensive than previously described in the literature or in any database.

The populations reported at the most extreme latitudes such as in Canada, Russia and parts of northern Europe are established exclusively in water bodies where the temperature is elevated due to geothermal or industrially-created heat. Although self-sustaining, these populations do not have any invasive potential as they will always be limited by temperature. Nevertheless, their success in such habitats demonstrates a remarkable opportunism, where they have colonised narrow bands of habitat in which environmental conditions allow their survival. Furthermore, it does not exclude the possibility of adverse impacts in these places [31].

Climate change is an important consideration regarding the future of the distribution of aquatic invasive species worldwide [32]. It is likely that the establishment, spread and general success of invasive species will increase because they tend to already have traits that make them better at adapting to a changing environment - such as broad environmental tolerances, short generation times and high rates of dispersal [33]. The guppy certainly possesses many of the physiological, behavioural and life-history characters that are associated with extreme adaptability [15], and it is clear that its current range is at least partly dictated by temperature constraints. Inevitable escapees and releases from the pet trade mean that the guppy is frequently being introduced to locations that are outside of its environmental tolerance range, but as water temperatures rise, an increasing number of these introductions may result in the establishment of self-sustaining populations [31].

Human commensalism has frequently been associated with the success of invasive species [34]. In the case of the guppy, its use by humans both as a popular pet and as a biological control agent has allowed it to be transported throughout the world, constantly providing opportunities for invading new habitats. A total of 115 species of invasive freshwater fish are already established worldwide as a result of the global aquarium trade, which continues to grow by 14% annually [26]. Fish introduced by this route tend to be healthy adults, who have often already been inadvertently selected for traits such as hardiness (i.e. to have greater physiological tolerance to changes in water quality and temperature) by the domestication or transportation process [26], [35].

As with many threats to biodiversity, the problem of invasive guppies is largely restricted to the regions that are least well equipped in terms of scientific and financial resources and therefore very little is done in terms of impact assessment. At the same time, the control of mosquito-borne disease continues to be of utmost importance in many of these countries, and poeciliids such as guppies are frequently seen as a cheap and easy potential solution [36]. Our survey brought together many reports of the destructive impact of guppies on a range of native species and ecosystems worldwide, the majority of which are anecdotal. Determining whether a species has *caused* a reduction in diversity or whether they are simply better at colonising those habitats which are already depauperate of native fish is extremely difficult. Fears of the effects of guppies on native species, coupled with their expanding range due to human intervention, suggests an urgent need for properly controlled studies.

### II. Mechanisms of establishment

We found that female guppies are capable of routinely establishing new populations in mesocosms, and that over 80% of these populations persist for at least two years. Moreover, and contrary to our predictions based on life-history differences, populations founded by females from the low predation localities were just as successful as those founded by females from high predation localities. It is important to remember that the two source populations of the founders of the mesocosm populations express the natural range of life history variables in native populations. This suggests that the origin of wild-type guppies has little bearing on the likelihood that a successful population will be established. Although there were initial differences in brood size in line with previous studies [29], in the direction that female founders sourced from high risk habitats have larger broods, these appear to have little bearing on establishment probability or population size after one or two years.

We also found no significant differences in performance between the offspring of wild and mesocosm fish, within each of the two founder populations, suggesting that behavioural viability is maintained in populations founded by a single female, at least for the duration of this experiment. It is not possible to know the exact pedigree of the fish tested, which is likely to be complicated and cross-generational. However, assuming a three month maturation period and a one month gestation period [15], they would have most likely been between three and six generations from the founder. Severe demographic bottlenecks such as those manipulated in this study are likely to be commonplace in introduction scenarios [37], thus it is of great interest that these events do not necessarily reduce colonisation ability or the behavioural viability of resulting populations.

There are a number of studies that have examined the effect of demographic bottlenecks on the genetics of poeciliid populations, both in the context of experimental manipulations [38], [39], as well as in relation to native [21], [40] and feral [37], [41], [42] populations. When very small founder numbers (<10) are involved, bottlenecks are almost always detectable using molecular markers, which reveal reductions in allele frequency and heterozygosity [21], [38], [39]. Studies of introduced populations produce mixed findings; in some cases bottlenecks are revealed [37], [42], and in other cases there is little evidence of founder effects [41]. Introduced populations of poeciliids, particularly those originating from unwanted pets, or from fish placed in a water tank to control mosquito larvae, are likely to have descended from very few founding individuals. Consequently, demographic bottlenecks may be a common occurrence in the evolutionary history of non-native poeciliid populations. Thus far, most studies have used molecular approaches to detect changes in neutral genetic variation following bottleneck events; here we examined their effects on phenotypic traits. In the context of introduced populations, differences in phenotypic traits are likely to be more important to invasive success than the extent of neutral genetic variation [37].

It is striking that behavioural performance was not impaired in our mesocosm fish, especially given that other studies have detected behavioural evidence of inbreeding in guppies [43]. The guppy has been shown to employ a number of pre- and post-copulatory strategies that could help to minimise inbreeding [44], [45]. By encouraging the fertilisation success of the sperm of the least related males, females have the potential to produce less-inbred offspring. Even over several generations in our mesocosms, this could result in a considerably less inbred population when compared with a randomly fertilising population. Such a strategy may, at least partly, explain the lack of inbreeding effects seen here.

Nonetheless, the ancestral origin (i.e. whether fish were descended from high or low predation populations) had a significant effect on both evasion ability and reaction distance. Contrary to the pattern seen in adult fish from similar pairs of populations, where those who have evolved in low predation populations display less pronounced antipredator behaviours [27], the offspring in this study showed the reverse pattern; those descended from low predation populations displayed stronger antipredator behaviours than those descended from high predation fish. While the low predation locality supports fewer predators of adult guppies, the greater abundance of smaller predatory species such as *Rivulus hartii*, and possibly higher levels of cannibalism due to higher densities and larger adults [29], may lead to a stronger selective pressure on newborn antipredator behaviour here than in the ‘high predation’ location downstream. Previous work has shown that there can be a strong shoaling tendency amongst newborn guppies in populations where adults shoal very little [46].

Life history traits have been linked to invasive success in a variety of taxa [47], [48]. McMahon [49] found that invasive bivalves in North America were characterised by rapid growth, early maturity and elevated fecundity, all of which encouraged rapid recovery after population reductions. Single-parent or vegetative reproductive strategies are commonly associated with invasive species for the same reason [47], [50]. For example, the water hyacinth *Eichhornia crassipes* has the most highly developed asexual reproduction strategy within its genus, and is also by far the most invasive [51]. Taylor & Hastings [12] suggested that this is partly because such strategies minimize Allee effects in small introduced populations, increasing their invasive potential. Sperm storage and the subsequent birth of live young can be viewed as a parallel strategy in guppies, enabling a succession of broods to be born without the need for further male contact [19].

The fish in our experiment were in single species assemblages, and at this stage we do not know if the same levels of population establishment and growth would be maintained in the light of competition or predation. Nonetheless, as noted earlier, guppies and other poeciliids are often introduced into low diversity localities that are remarkably similar to the mesocosms in this study. These include ponds or water tanks where guppies are used for malaria control [24], and where they may not encounter other species until their populations have substantially increased. This initial population growth will depend largely on juveniles successfully evading cannibalistic attacks by older individuals [30]. Cannibalism levels could be elevated in small, artificial water bodies – our own observations suggest that juvenile fish in containers lacking weed or other structure are particularly vulnerable. Here we have demonstrated that juvenile antipredator behaviours are indeed retained over several generations in this type of enclosed habitat, thus maintaining colonisation potential in common biological control scenarios.

Most species introduced to a new habitat will either fail to thrive or be unable to establish a self-sustaining population [52]. The documented success of introduced poeciliid fish worldwide, however, suggests that this family of freshwater fish is particularly well suited to doing both of these things. Of 20 poeciliid species recorded as having been introduced outside of their native range, 18 of them are listed as ‘established’ or ‘probably established’ in at least one country [16] and together they are responsible for 11% of fish species on the Global Invasive Species Database, including being represented by *Gambusia affinis* on their list of ‘One Hundred of the World's Worst Invasive Alien Species’ [14]. Indeed, poeciliids possess many of the traits associated with invasive success [47], most notably phenotypic plasticity [21], polyphagy [53]), eurytopy [54], and ovoviviparity [18]. The remarkable establishment success demonstrated in this study, which was independent of the origin of the founding females, emphasises the critical importance of the latter. It is also important to remember that although these fish may be initially contained within water tanks or pools, it is likely that these will overflow, for example during heavy rains, or be washed out by householders, or that juvenile guppies will escape through outflows. Once the fish are established in the wild, it may be very difficult to eradicate them [55].

Our results demonstrate how introductions consisting of a few animals, or even a single individual, can lead to thriving populations of invasive species. A highly specialised reproductive system, coupled with a remarkable adaptability [27], [56] is likely to have led to the phenomenal success of the guppy outside of its native range. These findings reinforce the need for caution when releasing exotic species, and show that seemingly innocuous or beneficial activities such as a child freeing a few pet fish or a concerned householder using guppies to control mosquitoes can result in a thriving population of invasive poeciliids that may then go on to compete with the indigenous freshwater fauna. They also illustrate how many small actions replicated across the globe, in the form of the accidental or deliberate release of a few fish, combined with natural adaptations in these fish for life in ephemeral habitats, can contribute to the reduction of diversity in freshwater fish assemblages worldwide.

## Materials and Methods

### I. Worldwide distribution

An e-mail questionnaire (see Text S1) was sent to scientists working in universities, governmental organisations and non-governmental organisations worldwide. Recipients were selected primarily by conducting internet searches for key words and phrases such as “freshwater fish research” and the name of the country in question. Some were also found by searching the online scientific literature for similar key words and contacting authors. Others were suggestions made by existing contacts. A map displaying the locations of respondents was updated regularly, so that geographical gaps could be identified and areas with poor response rates specifically targeted.

The questionnaire provided data on:

- the presence, absence or unknown status of *Poecilia reticulata* in a specified region
- the year of first introduction, where known
- the purpose behind the introductions, where known
- reported negative effects of the introductions
- information on the distribution and origins of the introductions

Responses to the survey that reported the presence of guppies in a particular region were recorded on a spreadsheet, along with any additional information, and added to a GIS database (Manifold version 8).

Reported absences include only instances where researchers were confident that they have not come across the species when they would have expected to during their work or the work of others had it be present. Where the respondent was unsure or ‘unaware of presence’ this was not included as a negative data point.

Existing reports documenting guppy presence compiled by FishBase [16] were also included. Other databases such as that overseen by the United States Geological Survey [57], the Global Invasive Species Database [14], the Fisheries and Agriculture Organisation of the United Nations invasive species database [58] and the South African Biodiversity Information Facility [59] were consulted in conjunction with the questionnaire responses to help build up the most comprehensive picture of the worldwide distribution of the guppy to date.

### II. Mechanisms of establishment

#### Mesocosm set-up

Thirty plastic mesocosms (100 cm×40 cm×30 cm; water 20 cm deep), were placed on the rooftop at the University of the West Indies, St Augustine, in Trinidad. Gravel and vegetation (water hyacinth, *Eichhornia crassipes* and Canadian pondweed, *Elodea canadensis*) provided cover. Tanks were covered with wire mesh to prevent aerial predation and fish escape. One wild-caught female was introduced to each mesocosm. Half of these were from the Upper Tunapuna, a low predation river, the remainder from the Lower Tacarigua, a high predation river [60]. Guppy origin was alternated along the line of mesocosms. There was no significant difference in size (total length) between females from the two localities (t = 0.35; df = 28; p = 0.732; low predation females = 35.1 mm; high predation females = 35.4 mm; SE = 0.7 in both cases); Wild guppy females of this size are almost invariably pregnant and have stored sperm [19]. The mesocosms relied on natural productivity and were topped up with water when necessary. Water temperature ranged between 22–28°C. The experiment ran for two years from April 2007 to May 2009.

#### Assessment of performance

All mesocosm fish were caught, counted and measured at 12 and 24 months. Females measuring >16 mm were considered to be sexually mature [29]; these were isolated in individual containers and checked for offspring several times daily. This generated newborns for the performance tests. Remaining fish were returned to their respective mesocosms after the census. Wild-caught females from both original sites were isolated in the same manner. Containers were labelled according to an arbitrary code with corresponding key to enable ‘blind’ testing. After giving birth, females were removed, re-measured and returned to their mesocosm or wild stock tank. Schooling, evasion ability, time in cover, activity and reaction distance were assessed in newborn fish. Pairs of newborns were transferred to a circular white tray (30 cm diameter; water 2 cm deep) and left to settle for 5 min. The ‘*schooling*’ behaviour of the focal individual (that is the time it spent swimming within 3.5 body lengths of its companion [61]) was then recorded for 5 min. *Evasion ability* was assessed as the time taken to capture an individual using a small (3 cm) net presented in a standard fashion [61]. The remaining behaviours were recorded for single individuals placed in small white arenas (21×15×8 cm deep, water depth 2.5 cm). In the first set of trials the arena was split into four quadrants, which were alternately either gravel-covered or open. Gravel was used because it provides potential refuge, both in the form of physical places to hide and camouflage. *Time in cover* (out of 5 min) was the time spent in the gravel zone while *activity* was the number of movements between zones. The final trials recorded *reaction distance* and were conducted in a gravel-free arena. This was the distance at which an individual responded to a looming object (a black pencil) that moved towards it at a speed of 2.5 mm/second.

82 broods were tested for evasion ability, time in cover, activity and reaction distance; 58 were additionally tested for schooling. Fewer trials were possible for schooling, as pairs of fish were required. In some cases where broods were large, a subset of 6–8 newborns was tested.

#### Statistical analysis

All statistical tests were performed using SPSS v.17.0.0. Population sizes were compared using a repeated measures ANOVA, with year as the repeated variable. The mean value per brood was used in all behavioural analyses. No significant differences were found between populations aged 12 and 24 months for any behaviour, therefore data from both were combined and compared with the data from wild fish. These behavioural data were analysed using a two-way MANOVA, examining the effect of origin of mother (wild or mesocosm) and evolutionary history (high predation or low predation). As the interaction term was not significant (F_5,46_ = 1.127; p = 0.360), the model was re-run without it. A principal components analysis was used to provide an integrated measure of performance and as both PC1 and PC2 each explained more than 25% of the variance, the scores for these components were analysed using a two-way ANOVA. As before, the interaction terms were not significant (PC1: F_1,74_ = 0.569; p = 0.453; PC2: F_1,74_ = 0.152; p = 0.689) and were removed from the models. All data displayed a normal distribution and homogeneity of variance, with the exception of *time in cover* where data were squared in order to meet these assumptions.

#### Ethics

All animal work was conducted according to the relevant national and international guidelines. No aspect of this study required special approval from a committee; no animals were harmed or killed and no invasive methods were used.

## Supporting Information

Text S1 **Email questionnaire.**
(DOCX)

Text S2 **Supplementary acknowledgments.**
(DOCX)

Figure S1 **Net evasion time and test order of trials.**
(TIF)

Table S1 **List of countries reporting the presence or absence of guppies.**
(DOCX)

Table S2 **Reports of the effects of guppies worldwide.**
(DOC)
