# Supplementary material for: How Reproductive Ecology Contributes to the Spread of a Globally Invasive Fish
Source: PLoS One. 2011 Sep 19;6(9):e24416. doi: 10.1371/journal.pone.0024416 (PMC3176282; doi:10.1371/journal.pone.0024416)
Supplement: Text S1 — Email questionnaire. (DOCX) [file pone.0024416.s001.docx]

**Text S1:** Email questionnaire

Subject: Invasive Guppies: Worldwide Survey

Dear [insert name],

We are hoping you might be able to help us with our work on invasive freshwater fish.

Specifically, we are keen to document the distribution and origin of invasive guppies (*Poecilia reticulata*) worldwide. Although this freshwater species is native to Trinidad and northern South America, it is now found in every continent – with the exception of Antarctica. They have been introduced for a variety of reasons, including their deliberate introduction as mosquito control agents and their incidental introduction as unwanted aquaria fish.

Despite much being known about the fish themselves, very little is known about the origins and reasons behind the introductions and the spread of these invasive species. We hope to construct a comprehensive database combining information from around the world to enhance our understanding of invasive guppies, and of invasive species in general.

We would be extremely grateful if you could spare a few minutes to answer the following questions:

1)        Are you aware of the presence of guppies in streams, rivers and ponds in [insert country/region]? (If not, please proceed to question 5). If possible, please specify regions or particular locations.

2)        If so, are the fish found throughout the region, or in localised parts only?

3)        Do you know anything about the origin of the introduction(s)? For example, when, where and/or why they were introduced?

4)        Are you aware of any negative effects on habitats, ecosystems or other fauna in areas where the fish are found (e.g. predation on/competition with native species)?

5)        Can you think of any other information regarding guppies in your region that might be of interest to us?

6)        Finally, if you can suggest any useful contacts that may help us with our enquiries we would be very grateful if you could let us know their name and/or contact details.

Our goal is to produce an online map showing the contemporary distribution of guppies and we will of course acknowledge your help in contributing records (including negative ones) to it.


Many thanks for your help,
