# Supplementary material for: How Reproductive Ecology Contributes to the Spread of a Globally Invasive Fish
Source: PLoS One. 2011 Sep 19;6(9):e24416. doi: 10.1371/journal.pone.0024416 (PMC3176282; doi:10.1371/journal.pone.0024416)
Supplement: Text S2 — Supplementary acknowledgments. (DOCX) [file pone.0024416.s002.docx]

**Text S2:** Supplementary acknowledgments

The authors wish to acknowledge and thank the following respondents for taking the time to provide us with information regarding the status of *Poecilia reticulata* worldwide. They are listed here by region (with the country about which they were able to supply information included in parentheses):

**Americas (including the Caribbean)**

Enrique Barraza (El Salvador), Roland Bucher (Peru), Paulo Buckup (Brazil), William Bussing (Costa Rica), Daniel Carnevia (Uruguay), Salvador Contreras-Balderas (Mexico)

Walter Courtenay (USA), Brian Dyer (Chile), Ronald Escoto (Nicaragua), Peter Esselman (Belize), Angela Fields (Barbados), Gonzalo Flores (Bolivia), Eric Hyslop (Jamaica), Licenciado Jose Arturo Nunez (El Salvador), Tom Kwak (Puerto Rico), Brian Langerhans (Bahamas, Cayman Islands, Dominican Republic, Jamaica, Puerto Rico, Uganda), Bill Loftus (US Virgin Islands, USA), Licenciado Lorenzo Becerra (Panama), Richard MacKenzie (Hawaii, Micronesia), Anne Magurran (Brazil), Wilfredo Matamoros (Honduras), William McLarney (Costa Rica), Danilo Mrdak (Montenegro), Hernan Ortega (Peru), Pablo Ribla (Argentina, Paraguay), Donald Stewart (Dominica, Ecuador)

Juan Schmitter –Soto (Mexico), Paul Shafland (USA), Michael Tobler (Mexico, USA).

**Oceania (Australasia and Pacific islands)**

Leon Barmuta (Australia), David Boseto (Australia, Cook Islands, Fiji, French Polynesia, Guam, New Caledonia, Palau, Samoa), Rob Brooks (Australia), Terry Donaldson (Guam), Tim Farrell (Australia), Jon Harding (Tonga), Aaron Jenkins (Fiji, New Caledonia, Vanuatu), Michael Jennions (Australia), Phillipe Keith (French Polynesia, New Caledonia, Reunion), Helen Larson (Australia), Gerald McCormack (Cook Islands), Bob McDowall (New Zealand), Steve McKagan (Marianas), Brad Pusey (Australia), Graham Pyke (Australia), Grant Scurr (Australia), Brent Tibbatts (Guam, Marianas, Micronesia, Palau), Kevin Warburton (Australia).

**Europe**

Vitor Almada (Portugal), Marcello Bazzanti (Italy), Elsa Brokkelkamp (Netherlands), Karim Erzini (Portugal), Emili Garcia-Berthou (Spain), Vlado Kovac (Slovakia), Constantinos Moustakas (Cyprus), Henrik de Nie (Netherlands), Carsten Nowak (Germany), Andrea Pilastro (Italy), Felipe Ribeiro (Lisbon), Marco Seminara (Italy), Ricardo Serrao Santos (Portugal), Skuli Skulason (Iceland), Lorenzo Tancioni (Italy), Leonidas Varnakas (Greece), Paul Veenvliet (Austria, Hungary, Netherlands. Slovakia, Slovenia).

**Africa (and West Indian Ocean islands)**

Chandani Abbadoo (Mauritius), Abdelhamid Azeroual (Morocco), Sixte Blanchy (Comoros), Rob Britton (Kenya), Hederick Dankwa (Ghana), Anis Diallo (Senegal),

Abebe Getahun (Ethiopia), Emily Hardman (Rodrigues), Carl Hopkins (Gabon), Cyprion Katongo (Zambia), Paul Loiselle (Madagascar, Uganda), Steven Lowe (South Africa)

Kit Magellan (South Africa), Victor Mamonekene (Democratic Republic of Congo, Republic of the Congo), Sean Marr (South Africa), Moeketsi Mokati (Eritrea)

Daniel Okeyo (Namibia), Babatunde Olaosebikan (Nigeria), Abdel Rahman (Egypt)

Hasan Shakeel (Maldives), Paul Skelton (South Africa), Jos Snoeks (Algeria, Comoros, Democratic Republic of Congo, Kenya, Morocco, Namibia, Republic of the Congo, Senegal), George Turner (Malawi), Sylvester Bwaku Wandera (Uganda), Olaf Weyl (Mozambique).

**Asia**

Afshin Afzali (Iran), Amir Ahmad (Malaysia), Vijay Anand (India), Raymon van Anrooy (Kyrgyzstan), Michel Bariche (Lebanon), Nina Bogutskaya (Russia), Christine Casal (Philippines), Brian Coad (Iran, Iraq), Arik Diamant (Israel), David Dudgeon (Hong Kong), Guler Ekmekci (Turkey), Priyantha Epa (Sri Lanka), Mazlan Ghaffar (Malaysia),

Dani Golani (Israel), Menachem Goren (Israel), Rafael D Guerrero (Philippines),

Najam Ul Huda Khan (Pakistan), Reiji Masuda (Japan), Joie Matillano (Philippines), Mizuki Matsunuma (Japan), So Nam (Cambodia), Nonn Pativong (Thailand), Ramani Shirantha (Sri Lanka), Chen Xiao-Yong (China).
