# Supplementary figures and images for: How Reproductive Ecology Contributes to the Spread of a Globally Invasive Fish
Source: PLoS One. 2011 Sep 19;6(9):e24416. doi: 10.1371/journal.pone.0024416 (PMC3176282; doi:10.1371/journal.pone.0024416)

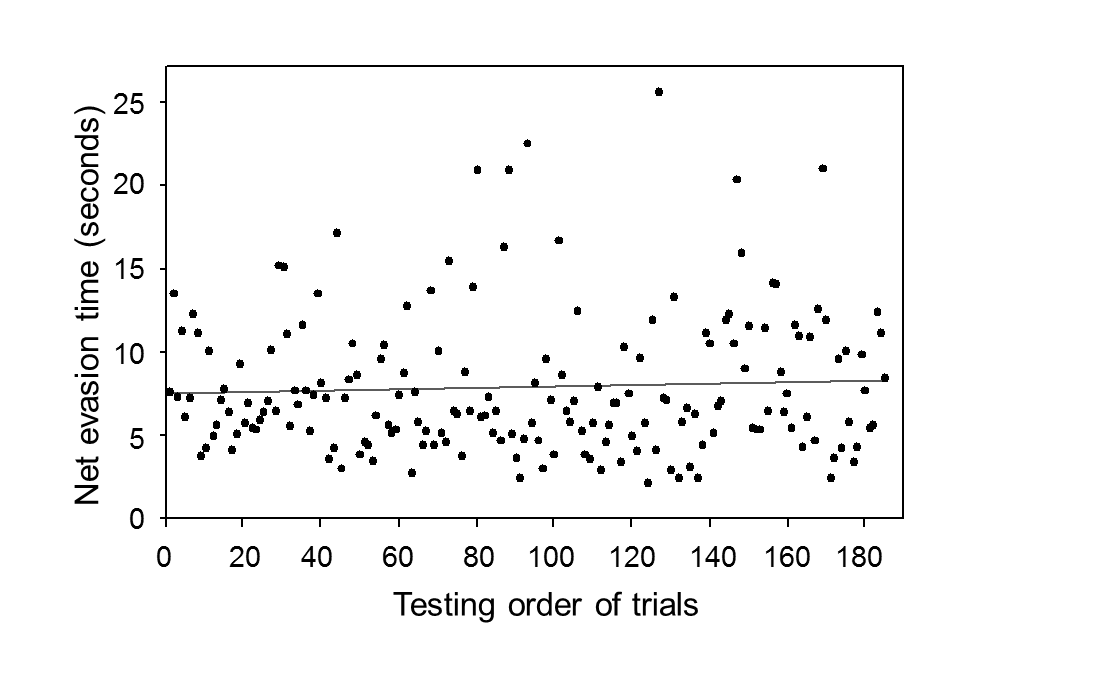

Supplement: Figure S1 — Net evasion time and test order of trials. (TIF) [file pone.0024416.s003.tif]
