# Supplementary material for: How Reproductive Ecology Contributes to the Spread of a Globally Invasive Fish
Source: PLoS One. 2011 Sep 19;6(9):e24416. doi: 10.1371/journal.pone.0024416 (PMC3176282; doi:10.1371/journal.pone.0024416)
Supplement: Table S1 — List of countries reporting the presence or absence of guppies. (DOCX) [file pone.0024416.s004.docx]

**Table S1:** List of countries reporting the presence or absence of guppies

| **Country** | **Guppies?** | **Date of first introduction** | **Purpose** |
| --- | --- | --- | --- |
| Albania | Yes |  |  |
| Algeria | Yes |  |  |
| Australia | Yes | 1960s | MC+AQ |
| Barbados | Yes | 1861 |  |
| Brazil | Yes | 1900s | MC+AQ |
| Cambodia | Yes |  | MC+AQ |
| Canada | Yes |  | AQ |
| Canary Islands | Yes |  |  |
| Colombia | Yes | 1940 | MC |
| Comoros | Yes | 1988 | MC |
| Cook Islands | Yes |  | MC |
| Costa Rica | Yes | 1962 | AQ |
| Cuba | Yes |  | MC |
| Czech Republic | Probably |  |  |
| DR of Congo | Yes | 1985 | MC |
| Dominica | Yes |  | MC+AQ |
| Dominican Republic | Yes |  |  |
| Fiji | Yes |  | AQ |
| French Polynesia | Yes |  |  |
| Germany | Yes |  | AQ |
| Ghana | Yes |  | MC |
| Guadeloupe | Yes |  | MC |
| Guam | Yes |  |  |
| Haiti | Yes |  |  |
| Hawaii | Yes | 1922 | MC |
| Hong Kong | Yes | 1940s | AQ |
| Hungary | Probably |  |  |
| India | Yes | 1908 | MC |
| Indonesia | Yes | 1920 | MC |
| Israel  Italy | Yes  Yes |  | AQ  AQ |
| Jamaica | Yes |  | AQ |
| Japan | Yes | 1974 | AQ |
| Kenya | Yes | 1956 | MC |
| Madagascar | Yes | 1960s | AQ |
| Malaysia | Yes |  | MC+AQ |
| Martinique | Yes |  |  |
| Mauritius | Yes | 1920 |  |
| Mayotte | Yes | 1980 | MC |
| Mexico | Yes |  | AQ |
| Morocco | Yes |  |  |
| Namibia | Yes |  | AQ |
| Netherlands | Yes |  | AQ |
| New Caledonia | Yes |  | AQ |
| New Zealand | Yes | 1920s |  |
| Nigeria | Yes | Colonial & 1972 | MC |
| Pakistan | Yes |  |  |
| Palau | Yes | 1914 | MC |
| Panama | Yes | 1900 | MC |
| Papua New Guinea | Yes | 1967 | AQ |
| Paraguay | Yes |  |  |
| Peru | Yes | 1940 | MC |
| Philippines | Yes | 1905 | MC+AQ |
| Puerto Rico | Yes | 1935 | AQ |
| Republic of the Congo | Yes |  |  |
| Reunion | Yes | 1950s | MC |
| Russia | Yes |  | AQ |
| Samoa | Yes |  |  |
| Saudi Arabia | Yes |  |  |
| Senegal | Yes |  | MC |
| Singapore | Yes | 1937 | MC+AQ |
| Slovakia | Yes | 1960s | AQ |
| South Africa | Yes | 1912 | MC+AQ |
| Spain | Probably |  |  |
| Sri Lanka | Yes | 1925 | MC |
| Taiwan | Yes |  | AQ |
| Thailand | Probably | 1948 |  |
| Uganda | Yes |  | MC |
| United Arab Emirates | Yes |  |  |
| US Virgin Islands | Yes |  | MC |
| USA | Yes | 1957 | MC+AQ |
| Vanuatu | Yes |  | AQ |
| Zambia | Yes | 1980 | AQ |
| Argentina | No |  |  |
| Azores | No |  |  |
| Bahamas | No |  |  |
| Bangladesh | No |  |  |
| Belize | No |  |  |
| Bolivia | No | 1970s | MC |
| Chile | No |  |  |
| Cyprus | No |  |  |
| Ecuador | No |  |  |
| Egypt | No |  |  |
| El Salvador | No |  |  |
| Eritrea | No |  |  |
| Gabon | No |  |  |
| Greece | No |  |  |
| Honduras | No |  |  |
| Iceland | No |  |  |
| Iran | No |  |  |
| Iraq | No |  |  |
| Italy | No |  |  |
| Kyrgystan | No |  |  |
| Malawi | No | 1980s | AQ |
| Maldives | No |  |  |
| Montenegro | No |  |  |
| Myanmar | No |  |  |
| Nicaragua | Possibly |  |  |
| Portugal | No |  |  |
| Rodrigues | No |  |  |
| Slovenia | No |  |  |
| Tasmania | No |  |  |
| Turkey | No |  |  |
| UK | No |  |  |
| Uruguay | No |  |  |

AQ=Aquarium releases; MC=Mosquito control.
