# Supplementary material for: How Reproductive Ecology Contributes to the Spread of a Globally Invasive Fish
Source: PLoS One. 2011 Sep 19;6(9):e24416. doi: 10.1371/journal.pone.0024416 (PMC3176282; doi:10.1371/journal.pone.0024416)
Supplement: Table S2 — Reports of the effects of guppies worldwide. (DOC) [file pone.0024416.s005.doc]

**Table S2:** Reports of the effects of guppies worldwide.

|  | **Negative effects** | **Other effects** | **Reference** |
| --- | --- | --- | --- |
| Kenya and Uganda | Decline in native cyprinodontid species. | Minimal resource overlap with other species in Lake Baringo, Kenya. |  |
| Madagascar | Decline in native fish *Pachyanchax sakaramyi* |  |  |
|  |  |  |  |
| Comoros |  | Effective in cisterns and basins, but not in wild. |  |
| Sri Lanka | Decline of native fish *Davario malabaricus* and native aquatic invertebrates. |  |  |
| Hong Kong | Decline of the native minnow, *Aphyocypris lini*. |  |  |
|  |  |  |  |
| India | Potential threat to Western Ghats fish fauna | Reduced Malaria in state of Karnataka. |  |
|  |  |  |  |
| Philippines | A pest in milkfish, *Chanos chanos*, ponds. |  |  |
| Papua New Guinea | Exclusion of native species from some streams. |  |  |
| Australia | Regarded as a pest |  |  |
|  |  |  |  |
| USA: Hawaii | Decline in native atyid shrimp, amphipod and Odonata species and the potential to spread of disease to native fish. Density of poeciliids positively correlated with nitrogen levels. |  |  |
| USA | Decline of native goodeid, *Crenichthys baileyi* in Nevada, and the Utah sucker, *Catostomas ardens* in Wyoming. Thought to spread of exotic trematodes. |  |  |
| Mexico | Decline in native goodeid species through competition and desert topminnows through spread of disease |  |  |
| Peru | Dietary overlap means have potential to displace native ichthyfauna |  |  |
| Brazil | Predictor of low native fish abundance and diversity |  |  |

**References**

1. Courtenay WR, Meffe GK (1989) Small fishes in strange places: a review of introduced poeciliids. In: Meffe GK, Snelson FF, editor. Ecology and Evolution of Livebearing Fishes (Poeciliidae). New Jersey: Prentice Hall. pp. 453.

2. Britton J, Jackson M, Muchiri M, Tarras-Wahlberg H, Harper D, et al. (2009) Status, ecology and conservation of an endemic fish, Oreochromis niloticus baringoensis, in Lake Baringo, Kenya. Aquatic Conservation: Marine and Freshwater Ecosystems 19: 487-496.

3. Loiselle P (2006) A review of the Malagasy Pachypanchax (Teleostei: Cyprinodontiformes, Aplocheilidae), with descriptions of four new species. Zootaxa 1366: 1-44.

4. Sabatinelli G, Blanchy, S., Majori, G., Papakay, M. (1991) Impact of the use of larvivorous fish Poecilia reticulata on the transmission of malaria in FIR of Comoros. Annales de parasitologie humaine et comparée 66: 84-88.

5. Shirantha RRAR, A. A. D. Amarathunga and K. A. W. S. Weerasekara Do feral populations of Poecilia reticulata ready to bring its overall efficacy as a mosquito bio-control agent or pose threats to aquatic biodiversity in Sri Lanka; 2008; Colombo, Sri Lanka. Sri Lanka Association for the Advancement of Science (Section D), National Science Foundation and Biodiversity Secretariat, Ministry of Environment and Natural Resources.

6. Man SH, and Hodgkiss, I.J. (1981) Hong Kong freshwater fishes. Hong Kong: Urban Council, Wishing Printing Company. 75 p.

7. Ghosh SK, Tiwari SN, Sathyanarayan TS, Sampath TRR, Sharma VP, et al. (2005) Larvivorous fish in wells target the malaria vector sibling species of the Anopheles culicifacies complex in villages in Karnataka, India. Transactions of the Royal Society of Tropical Medicine and Hygiene 99: 101-105.

8. Raghavan R, Prasad, G., Anvar-Ali, P. H. and Pereira, B. (2008) Exotic fish species in a global biodiversity hotspot: observations from River Chalakudy, Western Ghats, Kerala, India. Biological Invasions 10: 37-40.

9. Froese R, Pauly D (2010) FishBase.

10. Juliano RO, Guerrero III, R. and Ronquillo, Inocencio. The introduction of exotic aquatic species in the Philippines. In: De Silva SS, editor. Proceedings of the workshop on introduction of exotic aquatic organisms in Asia.; 1989; Manila, Philippines. Asian Fisheries Society. pp. 154 (183-190).

11. Allen GR (1991) Field guide to the freshwater fishes of New Guinea. Singapore: Christensen Research Institute. 268 p.

12. Arthington A (1989) Diet of Gambusia affinis holbrooki, Xiphophorus helleri, X. maculatus and Poecilia reticulata (Pisces: Poeciliidae) in Streams of Southeastern Queensland, Australia. Asian Fisheries Science 2: 193-212.

13. Englund RE (1999) The Impacts of Introduced Poeciliid Fish and Odonata on the Endemic Megalagrion (Odonata) Damselflies of Oahu Island, Hawaii. Journal of Insect Conservation 3: 225-243.

14. Eldredge LG (2000) Non-indigenous freshwater fishes, amphibians and crustaceans of the Pacific and Hawaiian islands. In: Sherley G, editor. Invasive species of the Pacific: A technical review and draft regional strategy. Samoa: South Pacific Regional Environment Programme.

15. Font WF, Tate DC (1994) Helminth parasites of native Hawaiian freshwater fishes: An example of extreme ecological isolation. The Journal of Parasitology 80: 682-688.

16. Brock RE, and Kam, A. K. H. (1997) Biological and water quality characteristics of anchialine resources in Kaloko-Honokohau National Historical Park, Honolulu. Cooperative National Park Resources Study Unit, University of Hawaii. 112 p.

17. MacKenzie RF, and Bruland, G. (2009) Fish community structure in Hawaii’s coastal wetlands. Hawaii Wetland Monitor: Hawaii Wetland Joint Venture.

18. Leberg PL, Vrijenhoek RC (1994) Variation among desert topminnows in their susceptibility to attack by exotic parasites. Conservation Biology 8: 419-424.

19. Valero A, Macias Garcia, C. and Magurran, A.E. (2008) Heterospecific harassment of native endangered goodeids by invasive guppies in Mexico. Biology Letters 4: 149-152.

20. Ortega H, Guerra H, Ramirez R (2007) The introduction of nonnative fishes into freshwater systems of Peru. In: Bert TM, editor. Ecological and Genetic Implications of Aquaculture Activities. pp. 247-278.

21. Ferreira CP, and, L. Casatti (2006) Integridade biotica de um corrego na bacia do Alto Rio Parana avaliada por meio da comunidade de peixes. Biota Neotropica 6: 1-25.
